# Effect of low-density lipoprotein level and mortality in older incident statin-naïve hemodialysis patients

**DOI:** 10.1186/s12882-023-03337-5

**Published:** 2023-10-02

**Authors:** Je Hun Song, Eun Hee Park, Jinsuk Bae, Soon Hyo Kwon, Jang-Hee Cho, Byung Chul Yu, Miyeun Han, Sang Heon Song, Gang-Jee Ko, Jae Won Yang, Sungjin Chung, Yu Ah Hong, Young Youl Hyun, Eunjin Bae, In O. Sun, Hyunsuk Kim, Won Min Hwang, Sung Joon Shin, Woo Yeong Park, Hyoungnae Kim, Kyung Don Yoo

**Affiliations:** 1 Division of Nephrology, Department of Internal Medicine, Ulsan University Hospital, University of Ulsan College of Medicine, 25 Daehakbyeongwon-Ro, Dong-Gu, Ulsan, Republic of Korea 44030; 2 Division of Nephrology, Department of Internal Medicine, Soonchunhyang University Seoul Hospital, 59 Daesagwan-Ro, Yongsan-Gu, Seoul, Republic of Korea 04401; 3 Division of Nephrology, Department of Internal Medicine, Kyungpook National University Hospital, Kyungpook National University School of Medicine, Daegu, Republic of Korea; 4 Division of Nephrology, Department of Internal Medicine, Soonchunhyang University Bucheon Hospital, Bucheon, Republic of Korea; 5 Division of Nephrology, Department of Internal Medicine, National Medical Center, Seoul, Republic of Korea; 6 Division of Nephrology, Department of Internal Medicine, Pusan National University Hospital, Pusan National University School of Medicine, Busan, Republic of Korea; 7 Division of Nephrology, Department of Internal Medicine, Korea University Guro Hospital, Korea University College of Medicine, Seoul, Republic of Korea; 8 Division of Nephrology, Department of Internal Medicine, Yonsei University Wonju College of Medicine, Wonju, Republic of Korea; 9 Division of Nephrology, Department of Internal Medicine, Yeouido St. Mary’s Hospital, College of Medicine, The Catholic University of Korea, Seoul, Republic of Korea; 10 Division of Nephrology, Department of Internal Medicine, Daejeon St. Mary’s Hospital, College of Medicine, The Catholic University of Korea, Seoul, Republic of Korea; 11 Division of Nephrology, Department of Internal Medicine, Kangbuk Samsung Hospital, Sungkyunkwan University School of Medicine, Seoul, Republic of Korea; 12 Division of Nephrology, Department of Internal Medicine, Gyeongsang National University Changwon Hospital, Changwon, Republic of Korea; 13 Division of Nephrology, Department of Internal Medicine, Presbyterian Medical Center, Jeonju, Republic of Korea; 14 Division of Nephrology, Department of Internal Medicine, Hallym University Chuncheon Sacred Heart Hospital, Hallym University College of Medicine, Chuncheon, Republic of Korea; 15 Division of Nephrology, Department of Internal Medicine, Konyang University Hospital, Daejeon, Republic of Korea; 16 Division of Nephrology, Department of Internal Medicine, Dongguk University Ilsan Hospital, Dongguk University School of Medicine, Goyang, Republic of Korea; 17 Division of Nephrology, Department of Internal Medicine, Keimyung University Dongsan Hospital, Keimyung University School of Medicine, Daegu, Republic of Korea; 18 Basic-Clinical Convergence Research Institute, University of Ulsan, 25 Daehakbyeongwon-Ro, Dong-Gu, Ulsan, 44030 Korea

**Keywords:** Low-density lipoproteins, Hemodialysis, Statins, Chronic kidney disease, All-cause mortality

## Abstract

**Background:**

This study aimed to analyze low-density lipoprotein cholesterol (LDL-C) levels and their relationship with mortality in order to identify the appropriate lipid profile for older Korean hemodialysis patients.

**Methods:**

We enrolled a total of 2,732 incident hemodialysis patients aged > 70 years from a retrospective cohort of the Korean Society of Geriatric Nephrology from 2010 Jan to 2017 Dec, which included 17 academic hospitals in South Korea. Of these patients, 1,709 were statin-naïve, and 1,014 were analyzed after excluding those with missing LDL-C level data. We used multivariate Cox regression analysis to select risk factors from 20 clinical variables among the LDL-C groups.

**Results:**

The mean age of the entire patient population was 78 years, with no significant differences in age between quartiles Q1 to Q4. However, the proportion of males decreased as the quartiles progressed towards Q4 (*p* < 0.001). The multivariate Cox regression analysis, which included all participants, showed that low LDL-C levels were associated with all-cause mortality. In the final model, compared to Q1, the hazard ratios (95% confidence interval) were 0.77 (0.620–0.972; *p* = 0.027), 0.85 (0.676–1.069; *p* = 0.166), and 0.65 (0.519–0.824; *p* < 0.001) for Q2, Q3, and Q4, respectively, after adjusting for covariates, such as conventional and age-specific risk factors. The final model demonstrated that all-cause mortality increased as LDL-C levels decreased, as confirmed by a restrictive cubic spline plot.

**Conclusions:**

In older hemodialysis patients who had not previously received dyslipidemia treatment, elevated LDL-C levels were not associated with increased all-cause mortality. Intriguingly, lower LDL-C levels appear to be associated with an unfavorable effect on all-cause mortality among high-risk hemodialysis patients.

**Supplementary Information:**

The online version contains supplementary material available at 10.1186/s12882-023-03337-5.

## Background

Chronic kidney disease (CKD) is closely linked to an elevated risk of cardiovascular disease (CVD), spanning from early kidney damage to end-stage kidney disease (ESKD) [1]. Importantly, dyslipidemia plays a substantial role in exacerbating atherosclerotic cardiovascular (CV) mortality during stages three to five of CKD [2]. Additionally, patients with both CVD and CKD face higher mortality rates compared to those with CVD but normal renal function [3].

Prior studies have demonstrated that reducing low-density lipoprotein cholesterol (LDL-C) can effectively decrease the risks associated with coronary artery disease, revascularization, and ischemic stroke. Consequently, effective management of LDL-C levels is essential for minimizing CVD risk in patients with CKD [4]. However, in hemodialysis (HD) patients, statin therapy appears to reduce the risk of CV events and has a positive effect on serum albumin and C-reactive protein (CRP) levels, although it has no conclusive impact on all-cause or CVD mortality [5]. In HD patients, an inverse relationship exists between serum cholesterol levels and overall mortality risk among high-risk populations [6]. This observation implies that HD patients with hypocholesterolemia exhibit lower survival rates, suggesting that hypocholesterolemia may be a risk factor for intradialytic hypotension (IDH) [7, 8]. Another study identified a connection between total cholesterol levels and all-cause, as well as CVD, mortality risk in the context of inflammation and poor nutrition [9]. Although several randomized controlled trials (RCTs) conducted since 2000 reported that statin use reduced serum LDL-C levels in HD patients, it did not significantly impact mortality [10–12]. As a result, current clinical practice guidelines recommend against initiating dyslipidemia control agents after the onset of dialysis in the absence of atherosclerotic CVD [13–15].

The Kidney Disease Improving Global Outcomes (KDIGO) 2013 clinical practice guideline specifically advises against initiating statin/ezetimibe combination therapy in HD patients without evidence of atherosclerotic CVD [14]. For instance, the Heart and Renal Protection (SHARP) trial included dialysis patients and compared the administration of moderate statin therapy (simvastatin 20 mg per day plus ezetimibe) with a placebo, assessing the occurrence of major atherosclerotic events in patients with CKD. However, the findings were not statistically significant for dialysis patients [12]. In another trial, researchers compared the administration of rosuvastatin (10 mg) with a placebo control in HD patients, reporting no significant differences in CVD mortality [11]. Considering these observations, the present study’s importance may be further emphasized, as merely reducing LDL-C levels with statins did not lead to decreased mortality rates. In essence, this study highlights the significance of LDL-C levels in HD patients who were not treated with statin therapy. Perspectives on statin therapy usage in older adults vary across different guidelines [13–16]. A meta-analysis revealed that lipid-lowering interventions were equally effective in patients aged over 75 years compared to their younger counterparts [17].

The objective of this research was to examine LDL-C levels and their association with all-cause mortality in Korean dialysis patients aged 70 years and older. This study aimed to determine the potential applicability of the findings to older adults, in line with existing clinical practice guidelines.

## Materials and methods

### Study population

**Fig. 1:**
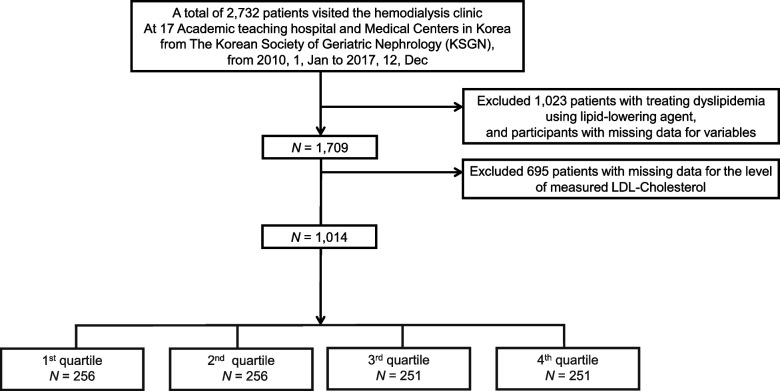
Study flow chart

Data from 2,732 incident HD patients aged ≥ 70 years who were registered through the Korean Society of Geriatric Nephrology (KSGN), which is situated at 17 university hospitals in Korea, between January 1, 2010, and December 31, 2017, were analyzed. Based on LDL-C levels, 1,014 patients were grouped into quartiles (Q1, Q2, Q3, and Q4) after exclusion of 1,023 patients who were taking a statin (Fig. 1). Statin use was defined as taking statins on the day closest to the date of HD initiation. Patients who started emergency HD due to acute kidney injury or peritoneal dialysis and those whose death information was absent were also excluded. Age, sex, body mass index (BMI), comorbidities [such as history of admission, diabetes mellitus (DM), CVD, hypertension, liver cirrhosis], malignancy, medication histories, cause of ESKD, and vascular access at the initial dialysis date were considered. Albumin, white blood cell (WBC) count, hemoglobin, high density lipoprotein cholesterol (HDL-C), LDL-C, triglycerides (TG), calcium, and phosphate were measured at the initiation of dialysis. In this study, only the measured LDL values were used, and calculated LDL values were not utilized due to the potential interference effects from triglycerides (TG). The Korean National Statistical Office (Microdata Integrated Service, on-demand, 20,180,619, https://mdis.kostat.go.kr) provided the mortality data until Dec 31, 2020.

All study methods were carried out in accordance with applicable guidelines and regulations. Patients’ clinical data were collected after receiving approval from the Institutional Review Board (IRB) for each study period and was carried out in accordance with the principles of the Helsinki Declaration. Each IRB did not require informed consent, and personally identifiable information was adequately protected. The following IRBs waived informed consent: Korea University Guro Hospital, Soonchunhyang University Seoul Hospital, The Catholic University of Korea, Incheon St. Mary’s Hospital, Hallym University Chuncheon Sacred Heart Hospital, Keimyung University Dongsan Hospital, Gyeongsang National University Changwon Hospital, Presbyterian Medical Center, Dongguk University Ilsan Hospital, WONJU SEVERANCE CHRISTIAN HOSPITAL, Ulsan University Hospital, Soonchunhyang University Bucheon Hospital, Yeouido St. Mary’s Hospital, Kyungpook National University Hospital, Pusan National University Hospital, Kangbuk Samsung Hospital, Konyang University Hospital, and Daejeon St. Mary’s Hospital. The IRB numbers for each institution involved in this study are provided in the supplementary materials (Table S1).

### Statistical analyses

Continuous and nominal variables are expressed as the mean and standard deviation, and those with normal distribution were analyzed using Student’s t-test, independent two-sample t-test, and analysis of variance. Those with abnormal distribution were analyzed with the Wilcoxon’s rank-sum test. Categorical variables are expressed as frequencies and percentages and analyzed with the Chi-squared or Fisher’s exact test. The Kaplan–Meier survival curve was used to examine differences in the survival rate of the LDL-C quartile groups. Cox proportional hazards models examined mortality based on LDL-C, TG, and HDL-C levels. Furthermore, the variance influence factor was used to confirm multicollinearity. Statistical significance was set at *P* < 0.05. All statistical analyses were carried out using SPSS software (version 21 IBM, Illinois) and the R programming language (version 4.2.2; R Foundation for Statistical Computing). In this study, we employed a restrictive cubic spline curve method using R software to analyze the relationship between variables. A restricted cubic spline function was fitted to the data to provide a flexible, non-linear representation of the association, allowing for a detailed examination of potential inflection points and changes in the slope. Knots were placed at predefined percentiles of the continuous predictor to create a smooth curve. The results were then interpreted visually, with the plotted curve illustrating the relationship between the variables under investigation.

## Results

### Baseline characteristics of the study population

Out of 2,732 patients, 1,041 statin-naïve participants were finally analyzed. After dividing them into quartiles based on LDL-C levels, the mean age at the beginning of dialysis was 78.3 ± 5.8, 77.9 ± 5.6, 78.1 ± 5.9, and 78.1 ± 5.6 years for Q1–Q4, respectively, with no significant difference between quartiles (*p* = 0.858). The prevalence of men in Q1–Q4 was 32.4%, 38.7%, 46.6%, and 51.4%, respectively (*p* < 0.001). However, there was no distinction between primary etiology, malignancy, metastatic malignancy, ischemic heart disease, cerebrovascular disease, congestive heart failure, atrial fibrillation, and DM, or the prevalence of hypertension and liver cirrhosis between groups. Furthermore, there was no difference in vascular access between the groups at the start of dialysis, nor was there a difference in albumin, hemoglobin, WBC, calcium, and phosphate levels. Additionally, there was no significant difference in medication history, such as ACE inhibitors and antiplatelet agents between the groups, except angiotensin receptor blockers (*p* = 0.044). Lastly, while there was a significant difference in use of maintenance dialysis access (*p* < 0.001), the proportion of patients undergoing maintenance dialysis with arteriovenous fistula (AVF) increased as the quartiles progressed towards Q4; however, this difference was not significant (Table 1).

**Table 1:** Baseline characteristics of the study population according to LDL-C level in statin naïve patients

Variables	Statin naïve					*p*-value
	Total (*N* = 1014)	Q1 (*N* = 256)	Q2 (*N* = 256)	Q3 (*N* = 251)	Q4 (*N* = 251)	
Age at the start of dialysis	78.1 ± 5.7	78.3 ± 5.8	77.9 ± 5.6	78.1 ± 5.9	78.1 ± 5.6	0.858
Survival duration (days)	1153.04 ± 947.27	962.27 ± 875.07	1220.31 ± 980.36	1126.66 ± 954.17	1306.65 ± 947.83	< 0.001
Sex						< 0.001
Female	428 (42.2%)	83 (32.4%)	99 (38.7%)	117 (46.6%)	129 (51.4%)	
Male	586 (57.8%)	173 (67.6%)	157 (61.3%)	134 (53.4%)	122 (48.6%)	
Primary etiology						0.093
Diabetic kidney disease	450 (44.4%)	122 (47.7%)	102 (39.8%)	112 (44.6%)	114 (45.4%)	
Glomerulonephritis	67 (6.6%)	12 (4.7%)	17 (6.6%)	17 (6.8%)	21 (8.4%)	
Reno-vascular disease	266 (26.2%)	56 (21.9%)	68 (26.6%)	69 (27.5%)	73 (29.1%)	
Other	224 (22.1%)	66 (25.8%)	66 (25.8%)	52 (20.7%)	40 (15.9%)	
NA	7 (0.7%)	0 (0.0%)	3 (1.2%)	1 (0.4%)	3 (1.2%)	
Malignancy						0.455
No	846 (83.4%)	203 (79.3%)	218 (85.2%)	207 (82.5%)	218(86.9%)	
Complete response	137 (13.5%)	41 (16.0%)	33 (12.9%)	38 (15.1%)	25 (10.0%)	
Keep on treatment	20 (2.0%)	8 (3.1%)	3 (1.2%)	4 (1.6%)	5 (2.0%)	
Palliative setting	11 (1.1%)	4 (1.6%)	2 (0.8%)	2 (0.8%)	3 (1.2%)	
Malignancy (metastasis)						0.096
No	825 (81.4%)	192 (75.0%)	210 (82.0%)	211 (84.1%)	212 (84.5%)	
Yes	25 (2.5%)	9 (3.5%)	5 (2.0%)	7 (2.8%)	4 (1.6%)	
NA	164 (16.2%)	55 (21.5%)	41 (16.0%)	33(13.1%)	35 (13.9%)	
Cardiovascular disease						0.381
No	824 (31.3%)	200 (78.1%)	210 (82.0%)	203 (80.9%)	211 (84.1%)	
Yes	190 (68.7%)	56 (21.9%)	46 (18.0%)	48 (19.1%)	40 (15.9%)	
Congestive heart failure						0.639
No	863 (85.1%)	224 (87.5%)	216 (84.4%)	210 (83.7%)	213 (84.9%)	
Yes	151 (14.9%)	32 (12.5%)	40 (15.6%)	41 (16.3%)	38 (15.1%)	
Atrial fibrillation						0.605
No	924 (91.1%)	232 (90.6%	231 (90.2%)	234 (93.2%)	227 (90.4%)	
Yes	90 (8.9%)	24 (9.4%)	25 (9.8%)	17 (6.8%)	24 (9.6%)	
Diabetes						0.457
No	471 (46.4%)	109 (42.6%)	127 (49.6%)	118 (47.0%)	117 (46.6%)	
Yes	543 (53.6%)	147 (57.4%)	129 (50.4%)	133 (53.0%)	134 (53.4%)	
Hypertension						0.656
No	123 (12.2%)	32 (12.5%)	35 (13.7%)	29 (11.6%)	27 (10.8%)	
Yes	890 (87.8%)	224 (87.5%)	221 (86.3%)	221 (88.4%)	224 (89.2%)	
NA	1 (0.1%)	0 (0.0%)	0 (0.0%)	1(0.1%)	0 (0.0%)	
Liver cirrhosis						0.167
No	979 (96.5%)	242 (94.5%)	247 (96.5%)	246 (98.0%)	244 (97.2%)	
Yes	35 (3.5%)	14 (5.5%)	9 (3.5%)	5 (2.0%)	7 (2.8%)	
ACE inhibitor						0.211
No	985 (97.1%)	250 (97.7%)	244 (95.3%)	244 (97.2%)	247 (98.4%)	
Yes	29 (2.9%)	5 (2.0%)	12 (4.7%)	7 (2.8%)	4 (91.6%)	
NA	1 (0.1%)	1 (0.4%)	0 (0.0%)	0 (0.0%)	0 (0.0%)	
ARB						0.044
No	538 (53.1%)	140 (54.7%)	145 (56.6%)	141 (56.2%)	112 (44.6%)	
Yes	475 (46.9%)	116 (45.3%)	111 (43.4%)	109 (43.8%)	139 (55.4%)	
NA	1 (0.1%)	0 (0.0%)	0 (0.0%)	1 (0.1%)	0 (0.0%)	
Antiplatelet agents						0.144
No	575 (56.7%)	162 (63.3%)	138 (53.9%)	140 (55.8%)	135 (53.8%)	
Yes	438 (43.2%)	93 (36.3%)	118 (46.1%)	111 (44.2%)	116 (46.2%)	
NA	1 (0.1%)	1 (0.4)	0 (0.0%)	0 (0.0%)	0 (0.0%)	
Start HD access						0.534
Catheter	857 (84.55)	219 (85.5%)	211 (82.4%)	206 (82.1%)	221 (88.0%)	
AVF	121 (11.9%)	30 (11.7%)	34 (13.3%)	33 (13.1%)	24 (9.6%)	
AVG	35 (3.5%)	7 (2.7%)	11 (4.3%)	11 (4.4%)	6 (2.4%)	
PD	1 (0.1%)	0 (0.0%)	0 (0.0%)	1 (0.4%)	0 (0.0%)	
Maintenance HD access						< 0.001
Catheter	216 (21.3%)	76 (29.7%)	53 (20.7%)	54 (21.5%)	33 (13.1%)	
AVF	627 (61.8%)	142 (55.5%)	154 (60.2%)	160 (63.7%)	171 (68.1%)	
AVG	151 (15.9%)	36 (14.1%)	54 917.6%)	37 (14.7%)	43 (17.1%)	
PD	2 (0.2%)	0 (0.0%)	2 (0.8%)	0 (0.0%)	0 (0.0%)	
NA	8 (0.8%)	2 (0.8%)	2 (0.8%)	0 (0.0%)	4 (1.6%)	
BMI	23.07 ± 4.11	22.81 ± 4.86	22.79 ± 3.67	23.33 ± 4.02	23.34 ± 3.76	0.257
History of hospitalization (within 6 months)						0.400
No	557 (54.9%)	131 (51.2%)	131 (51.2%)	141 (56.2%)	154 (61.4%)	
< 1 month	383 (37.8%)	105 (41.0%)	103 (40.2%)	93 (37.1%)	82 (32.7%)	
> 1 month	72 (7.1%)	20 (7.8%)	21 (8.2%)	16 (6.4%)	15 (6.0%)	
NA	2 (0.2%)	0 (0.0%)	1 (0.4%)	1 (0.4%)	0 (0.0%)	
Albumin (g/dL)	3.35 ± 0.58	3.241 ± 0.58	3.372 ± 0.56	3.453 ± 0.55	3.346 ± 0.59	< 0.001
WBC (/mm^3^)	8863.14 ± 5956.34	9337.53 ± 5635.75	8675.88 ± 5000.90	8634.94 ± 6755.65	8797.76 ± 6312.46	0.518
Hb (g/dL)	9.05 ± 1.72	9.15 ± 1.74	8.81 ± 1.68	9.04 ± 1.52	9.19 ± 1.90	0.059
Calcium	8.15 ± 0.95	8.19 ± 1.01	8.18 ± 0.93	8.20 ± 0.83	8.04 ± 0.99	0.196
Phosphate	4.98 ± 2.15	4.68 ± 1.67	5.03 ± 1.67	5.19 ± 3.26	5.01 ± 1.48	0.057
Total cholesterol (mg/dL)	149.29 ± 48.74	107.85 ± 26.90	131.32 ± 22.81	156.89 ± 24.85	204.23 ± 51.58	< 0.001
Triglycerides (mg/dL)	122.60 ± 78.02	112.62 ± 88.25	103.63 ± 48.47	123.49 ± 64.27	151.36 ± 94.64	< 0.001
HDL-C	38.38 ± 15.09	33.98 ± 16.98	38.64 ± 15.58	39.09 ± 13.18	42.02 ± 13.18	< 0.001
LDL-C	90.33 ± 39.87	47.48 ± 13.39	74.17 ± 6.48	97.80 ± 7.69	143.03 ± 34.13	< 0.001
TG/HDL-C	4.14 ± 7.55	5.86 ± 13.98	3.22 ± 2.59	3.61 ± 2.76	3.86 ± 3.55	< 0.001
Non-HDLC/HDL-C	3.19 ± 2.35	2.92 ± 3.39	2.78 ± 1.56	3.28 ± 1.50	3.81 ± 2.29	< 0.001

*LDL-C* low density lipoprotein cholesterol, *ACE* angiotensin-converting enzyme, *ARB* angiotensin receptor blocker, *AVF* arteriovenous fistula, *AVG* arteriovenous graft, *PD* peritoneal dialysis, *WBC* white blood cell, *Hb* hemoglobin, *HDL-C* high density lipoprotein cholesterol, *NA* not applicable

### Plasma lipid profile according to the LDL-C quartile in statin-naïve patients

The overall plasma LDL-C level was confirmed to be 90.33 ± 39.87 mg/dL, and levels in each quartile were 47.38 ± 13.39, 74.17 ± 6.48, 97.80 ± 7.69, and 143.03 ± 34.13 mg/dL in Q1–Q4, respectively (*p* < 0.001). For total cholesterol, HDL-C, and TG, the overall mean levels were 149.29 ± 48.74, 38.38 ± 15.09, and 122.60 ± 78.02 mg/dL, respectively, with significant differences between groups (*p* < 0.001). Levels gradually increased as the number of LDL-C ranks increased (Table 1).

### Analysis of all-cause mortality according to LDL-C quartile

**Fig. 2:**
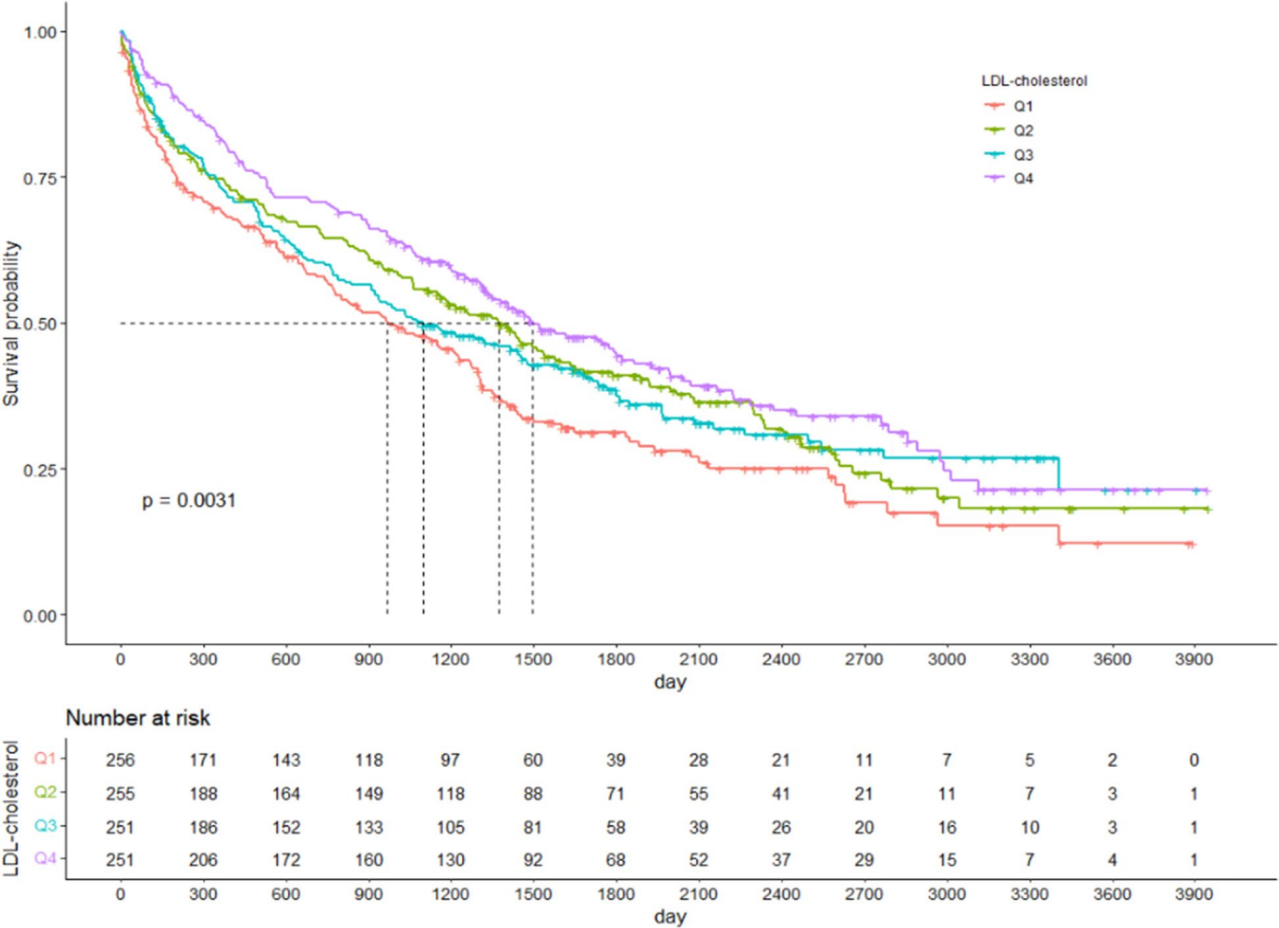
Comparison of patients’ survival according to the LDL-cholesterol level in the statin-naïve patients. A log rank test was used to compare the survival rates of each group based on their LDL-cholesterol level quartile. LDL, low density lipoprotein

**Fig. 3:**
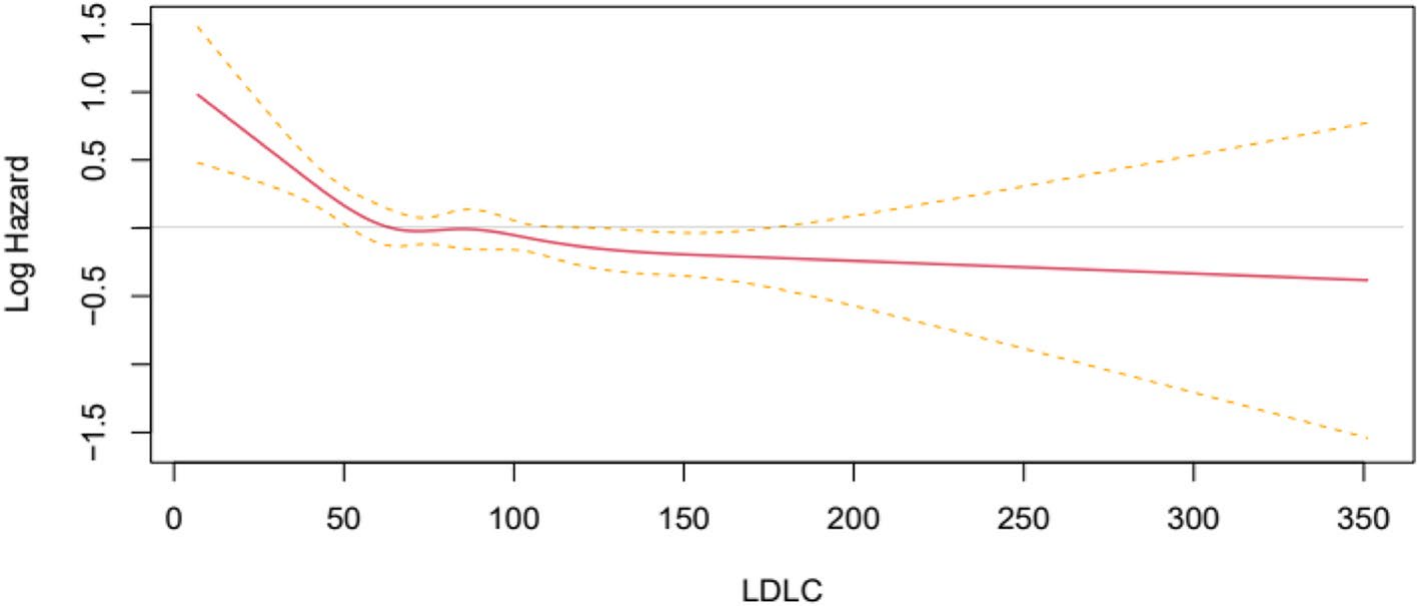
Non-linear association between the LDL-cholesterol level and mortality. A restrictive cubic spline is used to display the hazard ratio based on LDL-cholesterol level. LDL, low density lipoprotein

All-cause mortality was established using Cox regression and confirmed by dividing analyses into models 1, 2, and 3. Model 1 was crude, in model 2 we adjusted for sex and BMI, and in model 3 we adjusted for sex, BMI, serum albumin, concurrent history of hypertension and DM, liver cirrhosis, congestive heart failure, cerebrovascular accident, and history of hospitalization (within 6 months). The survival rate for each LDL-C quartile significantly differed based on the Kaplan–Meier curve analysis (Fig. 2). In statin-naïve patients, the hazard ratio (HR) decreased as the LDL-C level increased. This was especially the case after other covariates were adjusted in model 3. Compared to Q1, the HRs [95% confidence interval] were 0.77 [0.620–0.972; *p* = 0.027], 0.850 [0.676–1.069; *p* = 0.166], and 0.654 [0.519–0.824; *p* < 0.001] for Q2, Q3, and Q4, respectively. Furthermore, HRs increased significantly when the LDL-C level was less than 50 mg/dL using the restrictive cubic spline (Fig. 3).

## Discussion

We demonstrated that higher LDL-C levels are associated with a reduced risk of all-cause mortality in statin-naïve patients aged 70 years and older, initiating HD treatment in Korea, even after adjusting for covariates (Table 2). According to the multivariate cubic spline analysis, mortality increased as LDL-C levels decreased, and this trend became linear when LDL-C levels dropped below 50 mg/dL (Fig. 2).

**Table 2:** Hazard ratios of all-cause mortality according to LDL-C level (quartile)

	Statin naive		
	HR	95% CI	*P*
Model 1^a^			
Q2	0.779	0.630 – 0.965	0.022
Q3	0.803	0.647 – 0.996	0.046
Q4	0.664	0.533 – 0.827	< 0.001
Model 2^b^			
Q2	0.746	0.599 – 0.930	0.009
Q3	0.794	0.635 – 0.992	0.042
Q4	0.644	0.514 – 0.808	< 0.001
Model 3^c^			
Q2	0.777	0.620 – 0.972	0.027
Q3	0.850	0.676 – 1.069	0.166
Q4	0.654	0.519 – 0.824	< 0.001

*HR* hazard ratio, *LDL-C* low density lipoprotein cholesterol

^a^Model 1: crude

^b^Model 2: adjusted for sex, body mass index

^c^Model 3: adjusted for sex, body mass index, serum albumin, concurrent history of hypertension and diabetes mellitus, liver cirrhosis, congestive heart failure, cerebrovascular accident, history of hospitalization (within 6 months)

According to the review by Weiner et al., LDL-C levels exceed 130 mg/dL in 85% of patients with nephrotic syndrome, approximately 30% with CKD stage 5 HD, and 45% undergoing peritoneal dialysis[18]. Considering the context above, a study utilizing Korean claim data also indicates that the prevalence of dyslipidemia among Korean dialysis patients has been steadily increasing, approaching 60%. Given the continuous growth of the older dialysis patient population in Korea, understanding how to effectively manage lipids in these patients carries significant implications. In terms of LDL-C, as CKD progresses, the proportion of small, dense LDL-C increases, facilitating vessel wall infiltration and increasing susceptibility to oxidative modification, thereby increasing the proatherogenic effect [19]. As a result, an increase in LDL-C raises the risk of myocardial infarction. However, the increased risk of myocardial infarction following a decrease in estimated glomerular filtration rate (eGFR) tends to decline [20]. By this means, cholesterol and LDL levels can also reflect nutritional status; therefore, the intention of this study was to determine whether the “lower is better” proposition also holds true regarding incident dialysis for Korean older patients [21–24].

All prior RCTs and retrospective studies have focused on patients aged under 70 years, with no large-scale RCT conducted specifically in older dialysis patients to date. According to a meta-analysis, statin use effectively reduces LDL-C levels and major coronary events in the general older population, with effects observed across all age groups. However, the proportional risk reduction diminishes with age, leading to less favorable outcomes [25]. In contrast, a retrospective study using Health Insurance Review and Assessment service data from 65,404 Korean HD patients compared statin non-users before and after starting HD with those who initiated HD after using statins. Statin use for 3.6 years was associated with lower all-cause mortality, and this effect remained consistent even in patients aged 75 years and older [26]. Moreover, the Dialysis Outcomes and Practice Patterns Study, which included 7,635 HD patients, reported a 31% reduction in all-cause mortality (*p* < 0.001), a 23% reduction in CV mortality (*p* = 0.03), and a 44% reduction in non-CV mortality (*p* < 0.001) [27].

The reasons behind the lack of mortality impact in older dialysis patients, despite reduced LDL-C levels, remain inconclusive, and prior studies have yielded conflicting results. Factors such as inflammation and oxidative stress seem to have a more significant influence on mortality than do LDL-C levels. For instance, elevated CRP levels (> 5 mg/L) are associated with higher all-cause mortality and CVD in HD patients with type 2 DM, irrespective of their LDL-C levels [28]. Generally, inflammation induces endothelial changes that amplify inflammatory activity through various proinflammatory chemo-attractants, ultimately contributing to atherosclerosis [29]. The underlying mechanisms, however, differ between non-dialysis and dialysis patients with CKD. In patients with CKD, an increase in lipid peroxidation due to elevated levels of the nitric oxide synthase inhibitor, asymmetric dimethylarginine, leads to elevated CRP, oxidative stress, and endothelial dysfunction. These factors may contribute to an increased risk of CV mortality [30, 31].

In addition to these factors, impaired regulation of inflammatory transcription factors, such as p53 activator protein-1, signal transducer and activator of transcription, and nuclear factor-kB, may contribute to the complexity of the situation in patients with ESKD. Age-related increased oxidative susceptibility leads to constant genomic DNA damage, resulting in a pro-inflammatory state induced by altered systemic chemokine or cytokine activity [32]. Malnutrition is another contributing factor. In patients with ESKD, highly-sensitive CRP and interleukin-6 (IL-6) levels best predict malnutrition, while serum albumin, IL-6, and fetuin A levels best predict mortality [33]. Low albumin levels in patients receiving HD or peritoneal dialysis can lead to high all-cause mortality, which is related to malnutrition [34, 35]. In the presence of malnutrition, low total cholesterol levels increase mortality, while high total cholesterol levels without malnutrition also elevate mortality [9]. In our study, we observed that patients with low LDL-C levels tended to have low levels of serum albumin and phosphate, which may indicate malnutrition or an inflammatory state associated with an increased risk of mortality. It is crucial to note that this observation does not directly implicate statin use in increasing mortality risk. While statins have been associated with lowered cholesterol, it would be a leap to infer from the current data that they are responsible for inducing malnutrition or inflammation or that their use directly increases mortality risk in very-high-risk populations. The patients with low LDL levels seemed to have lower phosphate and albumin levels compared with the other quartiles; therefore, having low LDL levels appears to be a marker of malnutrition and inflammation. To this end, the results of subgroup analyses investigating the impact of LDL quartiles on mortality are presented in supplementary tables (Tables S2–S7). There could be numerous factors impacting the current outcomes, and it would be advantageous to investigate these further in a controlled study, accounting for other variables, such as diet, underlying health conditions, and other medication use.

As CKD progresses, arterial media calcification contributes to the rise in all-cause mortality and cardiovascular mortality [36]. In contrast, LDL-C levels determine the atherosclerotic plaque burden in the general population [37]. Consequently, arrhythmia (24.3%) is more common in patients with ESKD than atherosclerotic myocardial infarction (3.6%), and congestive heart failure (5.7%) is a cause of CV-related deaths [38]. CKD-MBD associated arteriosclerosis is the most influential factor here, followed by high systolic and low diastolic blood pressure [39]. Intradialytic hypotension (IDH) has been identified as an independent predictor of mortality [40] and myocardial and cerebral ischemia in HD patients, with hypocholesterolemia predicting a reduced vascular resistance response during IDH [8]. Levels of endothelin-1 (ET-1), the most important vasoconstrictor, are reduced when LDL-C levels are low due to endothelin-converting enzyme-1 inactivation [41]. Patients with low ET-1 levels also have significantly lower blood pressure during dialysis [42].

Many countries classify patients with advanced CKD as high-risk and recommend statin use; however, as previously mentioned, statin initiation is not recommended for dialysis patients. The findings of this study suggest that artificially lowering LDL-C levels in older, statin-naïve, dialysis patients could negatively affect all-cause mortality. Apart from statin use, novel medications, such as omega-3 fatty acids and Proprotein Convertase Subtilisin/Kexin type 9 (PCSK9) inhibitors, which are currently not recommended, can also lower LDL-C levels. For example, during daily administration of icosapent ethyl (4 g) to participants aged ≥ 50 years with a fasting triglyceride of 150–499 mg/dL and a median baseline eGFR of 75 mL/min/1.73 m^2^, both fatal and non-fatal ischemic CV events decreased compared to the placebo group [43]. Additionally, the Further Cardiovascular Outcomes Research with PCSK9 Inhibition in Subjects with Elevated Risk trial studied patients with clinical evidence of atherosclerosis and LDL-C levels of 70 mg/dL or non-HDL-C of 100 mg/dL. The effects of evolocumab were evaluated according to the stage of CKD. Regardless of CKD stage, the LDL-C lowering capacity and relative clinical efficacy and safety were consistent, and the risk of cardiovascular death, myocardial infarction, and stroke were reduced [44]. Although these novel medications are not yet widely used, further research may pave the way for their future application in dialysis patients.

This study has several limitations. First, due to the retrospective nature of this study, we could not completely rule out the possibility of selection bias. In addition, we were unable to investigate a specific reason why statins were not used. Second, analyses were solely conducted on data from Korea, which means that racial, regional, and sociocultural variations may have impacted the results and limited their generalizability. Additionally, as a retrospective cohort study, there exists uncertainty regarding causality, and time-dependent variables could not be verified until the time of death due to the reliance on laboratory findings at enrollment. Most importantly, the inability to determine the cause of death presents a significant limitation, as CV mortality could not be confirmed. Furthermore, the effects of novel medications, such as LDL-C lowering agents, omega-3 fatty acids, and PCSK9 inhibitors, have not yet been explored in this context, highlighting the need for more research in this area.

## Conclusion

In this study, while LDL-C levels serve as predictors of CVD in the general population, the relationship becomes more complex in HD patients, particularly in older, statin-naïve individuals initiating HD. Intriguingly, lower LDL-C levels appear to be associated with an unfavorable effect on all-cause mortality among a high-risk HD population.

### Supplementary Information

**Additional file 1:** **Table S1. **List of approval number for the research from the Institutional Review Board at each centre. **Table S2. **Hazard ratios of all-cause mortality by the sex according to LDL-C level (quartile). **Table S3. **Hazard ratios of all-cause mortality by the BMI according to LDL-C level (quartile). **Table S4. **Hazard ratios of all-cause mortality in DM according to LDL-C level (quartile). **Table S5. **Hazard ratios of all-cause mortality in albumin according to LDL-C level (quartile). **Table S6. **Baseline characteristics of the study population according to history for dyslipidemia medication. **Table S7. **Hazard ratios of all-cause mortality according to LDL-C level (quartile) using different reference cartegory. 

## Data Availability

The datasets used and/or analyzed during the current study are available from the corresponding author upon reasonable request.

## Abbreviations

BMI: Body mass index
CKD: Chronic kidney disease
CRP: C-reactive protein
CVD: Cardiovascular disease
CV: Cardiovascular
DM: Diabetes mellitus
eGFR: Estimated glomerular filtration rate
ESKD: End stage kidney disease
ET-1: Endothelin-1
HR: Hazard ratio
HD: Hemodialysis
HLD-C: High density lipoprotein cholesterol
IDH: Intradialytic hypotension
IL-6: Interleukin-6
LDL-C: Low-density lipoprotein cholesterol
PCSK9: Proprotein Convertase Subtilisin/Kexin type 9
RCTs: Randomized controlled trials
TG: Triglycerides
WBC: White blood cells
