# Supplementary material for: Effect of low-density lipoprotein level and mortality in older incident statin-naïve hemodialysis patients
Source: BMC Nephrol. 2023 Oct 2;24:289. doi: 10.1186/s12882-023-03337-5 (PMC10546714; doi:10.1186/s12882-023-03337-5)
Supplement: Supplementary file 1 — Additional file 1: Table S1. List of approval number for the research from the Institutional Review Board at each centre. Table S2. Hazard ratios of all-cause mortality by the sex according to LDL-C level (quartile). Table S3. Hazard ratios of all-cause mortality by the BMI according to LDL-C level (quartile). Table S4. Hazard ratios of all-cause mortality in DM according to LDL-C level (quartile). Table S5. Hazard ratios of all-cause mortality in albumin according to LDL-C level (quartile). Table S6. Baseline characteristics of the study population according to history for dyslipidemia medication. Table S7. Hazard ratios of all-cause mortality according to LDL-C level (quartile) using different reference cartegory. [file 12882_2023_3337_MOESM1_ESM.docx]

***Effect of Low-density Lipoprotein level and Mortality in Older Incident Statin-naïve Hemodialysis Patients***

**Authors**

**Je Hun Song^1,*^, Eun Hee Park^1,*^**, Jinsuk Bae^1^, Soon Hyo Kwon^2^ , Jang-Hee Cho^3^, Byung Chul Yu^4^, Miyeun Han^5^, Sang Heon Song^6^, Gang-Jee Ko^7^, Jae Won Yang^8^, Sungjin Chung^9^, Yu Ah Hong^10^, Young Youl Hyun^11^, Eunjin Bae^12^, In O Sun^13^, Hyunsuk Kim^14^, Won Min Hwang^15^, Sung Joon Shin^16^, Woo Yeong Park^17^, **Hyoungnae Kim^2^, Kyung Don Yoo^1,18^**, on behalf of the Korean Society of Geriatric Nephrology (KSGN)^19^

**Affiliations**

^1^Division of Nephrology, Department of Internal Medicine, Ulsan University Hospital, University of Ulsan College of Medicine, Ulsan, Republic of Korea

^2^Division of Nephrology, Department of Internal Medicine, Soonchunhyang University Seoul Hospital, Seoul, Republic of Korea

^3^Division of Nephrology, Department of Internal Medicine, Kyungpook National University Hospital, Kyungpook National University School of Medicine, Daegu, Republic of Korea

^4^Division of Nephrology, Department of Internal Medicine, Soonchunhyang University Bucheon Hospital, Bucheon, Republic of Korea

^5^Division of Nephrology, Department of Internal Medicine, National Medical Center, Seoul, Republic of Korea

^6^Division of Nephrology, Department of Internal Medicine, Pusan National University Hospital, Pusan National University School of Medicine, Busan, Republic of Korea

^7^Division of Nephrology, Department of Internal Medicine, Korea University Guro Hospital, Korea University College of Medicine, Seoul, Republic of Korea

^8^Division of Nephrology, Department of Internal Medicine, Yonsei University Wonju College of Medicine, Wonju, Republic of Korea

^9^Division of Nephrology, Department of Internal Medicine, Yeouido St. Mary's Hospital, College of Medicine, the Catholic University of Korea, Seoul, Republic of Korea

^10^Division of Nephrology, Department of Internal Medicine, Daejeon St. Mary's Hospital, College of Medicine, the Catholic University of Korea, Daejeon, Republic of Korea

^11^Division of Nephrology, Department of Internal Medicine, Kangbuk Samsung Hospital, Sungkyunkwan University School of Medicine, Seoul, Republic of Korea

^12^Division of Nephrology, Department of Internal Medicine, Gyeongsang National University Changwon Hospital, Changwon, Republic of Korea

^13^Division of Nephrology, Department of Internal Medicine, Presbyterian Medical Center, Jeonju, Republic of Korea

^14^Division of Nephrology, Department of Internal Medicine, Hallym University Chuncheon Sacred Heart Hospital, Hallym University College of Medicine, Chuncheon, Republic of Korea

^15^Division of Nephrology, Department of Internal Medicine, Konyang University Hospital, Daejeon, Republic of Korea

^16^Division of Nephrology, Department of Internal Medicine, Dongguk University Ilsan Hospital, Dongguk University School of Medicine, Goyang, Republic of Korea

^17^Division of Nephrology, Department of Internal Medicine, Keimyung University Dongsan Hospital, Keimyung University School of Medicine, Daegu, Republic of Korea

^18^Basic-Clinical Convergence Research Institute, University of Ulsan, Ulsan, Korea

^19^Korean Society of Geriatric Nephrology, Seoul, Republic of Korea

**^*^Je Hun Song, Eun Hui Park** equally contributed to this work as the first author

**Correspondence:**

**Kyung Don Yoo,** MD**,** PhD (Lead contact)
Associate Professor

Division of Nephrology, Department of Internal Medicine, Ulsan University Hospital, University of Ulsan College of Medicine

Basic-Clinical Convergence Research Institute, University of Ulsan

25 Daehakbyeongwon-ro, Dong-gu, Ulsan, Korea (44030)

Tel: 82-52-250-8658

E-mail: [ykd9062@gmail.com](mailto:ykd9062@gmail.com) , [ykd9062@uuh.ulsan.kr](mailto:ykd9062@uuh.ulsan.kr)

And

**Hyoungnae Kim,** MD, PhD

Assistant Professor

Division of Nephrology, Department of Internal Medicine, Soonchunhyang University Seoul Hospital, Soonchunhyang Medical College, Seoul, Republic of Korea 59 Daesagwan-ro, Yongsan-gu, Seoul, Korea, 04401 Fax; +82-2-709-9554

E-mail: [hkim@schmc.ac.kr](mailto:hkim@schmc.ac.kr)

**The Table of Contents for Supplemental Material**

**Table S1. List of approval number for the research from the Institutional Review Board at each centre**

|  | Author | Affiliation | IRB number |
| --- | --- | --- | --- |
| 1 | Gang-Jee Ko | Korea University Guro Hospital, Korea University College of Medicine | 2020GR0333 |
| 2 | Soon Hyo Kwon | Soonchunhyang University Seoul Hospital | SCHUH2020-01-011 |
| 3 | Dong Ryul Kim | The Catholic University of Korea, Incheon St. Mary's Hospital | OC20RIDI0015 |
| 4 | Hyunsuk Kim | Hallym University Chuncheon Sacred Heart Hospital, Hallym University College of Medicine | CHUNCHEON2020-02-002 |
| 5 | Woo Yeong Park | Keimyung University Dongsan Hospital, Keimyung University School of Medicine | DSMC 2020-01-036 |
| 6 | Eunjin Bae | Gyeongsang National University Changwon Hospital | GNUCH2019-12-021 |
| 7 | In O Sun | Presbyterian Medical Center | 2020-02-004 |
| 8 | Sung Joon Shin | Dongguk University Ilsan Hospital, Dongguk University School of Medicine | DUIH2020-02-019 |
| 9 | Jae Won Yang | Yonsei University Wonju College of Medicine | CR319174 |
| 10 | Kyung Don Yoo | Ulsan University Hospital, University of Ulsan College of Medicine | UUH2020-04-004 |
| 11 | Byung Chul Yu | Soonchunhyang University Bucheon Hospital | SCHBC 2020-01-009 |
| 12 | Sungjin Chung | Yeouido St. Mary's Hospital, College of Medicine, The Catholic University of Korea | SC20RIDI0011 |
| 13 | Jang-Hee Cho | Kyungpook National University Hospital, Kyungpook National University School of Medicine | KNUH2020-03-076 |
| 14 | Miyeun Han | Pusan National University Hospital, Pusan National University School of Medicine | H-2002-007-087 |
|  | Sang Heon Song |  |  |
| 15 | Young Youl Hyun | Kangbuk Samsung Hospital, Sungkyunkwan University School of Medicine | KBSMC2020-01-035 |
| 16 | Won Min Hwang | Konyang University Hospital | KYUH 2020-04-007 |
| 17 | Yu Ah Hong | Daejeon St. Mary's Hospital, The Catholic University of Korea | DC19RODI0088 |

**Table S2. Hazard ratios of all-cause mortality by the sex according to LDL-C level (quartile)**

|  | Statin naive | | | | | |
| --- | --- | --- | --- | --- | --- | --- |
|  | Male | | | Female | | |
|  | HR | 95% CI | *P* | HR | 95% CI | *P* |
| Model 1^†^ |  |  |  |  |  |  |
| Q2 | 0.815 | 0.626-1.063 | 0.132 | 0.719 | 0.500-1.034 | 0.075 |
| Q3 | 0.844 | 0.637-1.119 | 0.240 | 0.743 | 0.527-1.047 | 0.090 |
| Q4 | 0.679 | 0.506-0.911 | 0.009 | 0.636 | 0.452-0.895 | 0.009 |
| Model 2^‡^ |  |  |  |  |  |  |
| Q2 | 0.760 | 0.578-0.998 | 0.048 | 0.714 | 0.492-1.035 | 0.075 |
| Q3 | 0.838 | 0.627-1.120 | 0.233 | 0.743 | 0.523-1.056 | 0.098 |
| Q4 | 0.636 | 0.471-0.858 | 0.003 | 0.634 | 0.447-0.900 | 0.010 |
| Model 3^§^ |  |  |  |  |  |  |
| Q2 | 0.790 | 0.596-1.046 | 0.100 | 0.755 | 0.514-1.109 | 0.152 |
| Q3 | 0.917 | 0.682-1.232 | 0.566 | 0.757 | 0.527-1.089 | 0.133 |
| Q4 | 0.699 | 0.516-0.948 | 0.021 | 0591 | 0.412-0.848 | 0.004 |

^†^ Model 1: crude, ^‡^Model 2: adjusted for body mass index, ^§^Model 3: adjusted for body mass index, serum albumin, concurrent history of hypertension and diabetes mellitus, liver cirrhosis, congestive heart failure, cerebrovascular accident, History of hospitalization (within 6months)

HR = hazard ratio, LDL-C = Low density lipoprotein cholesterol

**Table S3. Hazard ratios of all-cause mortality by the BMI according to LDL-C level (quartile)**

|  | Statin naive | | | | | |
| --- | --- | --- | --- | --- | --- | --- |
|  | BMI < 25 | | | BMI >= 25 | | |
|  | HR | 95% CI | *P* | HR | 95% CI | *P* |
| Model 1^†^ |  |  |  |  |  |  |
| Q2 | 0.706 | 0.553-0.901 | 0.005 | 0.987 | 0.596-1.632 | 0.960 |
| Q3 | 0.701 | 0.544-0.903 | 0.006 | 1.209 | 0.753-1.942 | 0.431 |
| Q4 | 0.581 | 0.451-0.749 | <0.001 | 0.967 | 0.591-1.582 | 0.894 |
| Model 2^‡^ |  |  |  |  |  |  |
| Q2 | 0.719 | 0.563-0.918 | 0.008 | 0.948 | 0.570-1.577 | 0.839 |
| Q3 | 0.737 | 0.571-0.953 | 0.019 | 1.112 | 0.685-1.803 | 0.666 |
| Q4 | 0.603 | 0.467-0.778 | <0.001 | 0.874 | 0.528-1.447 | 0.600 |
| Model 3^§^ |  |  |  |  |  |  |
| Q2 | 0.728 | 0.566-0.936 | 0.013 | 1.093 | 0.650-1.838 | 0.736 |
| Q3 | 0.769 | 0.591-1.000 | 0.050 | 1.357 | 0.828-2.224 | 0.224 |
| Q4 | 0.592 | 0.456-0.768 | <0.001 | 1.082 | 0.641-1.826 | 0.767 |

^†^ Model 1: crude, ^‡^Model 2: adjusted for sex, ^§^Model 3: adjusted for sex, serum albumin, concurrent history of hypertension and diabetes mellitus, liver cirrhosis, congestive heart failure, cerebrovascular accident, History of hospitalization (within 6months)

HR = hazard ratio, LDL-C = Low density lipoprotein cholesterol

**Table S4. Hazard ratios of all-cause mortality in DM according to LDL-C level (quartile)**

|  | Statin naive | | | | | |
| --- | --- | --- | --- | --- | --- | --- |
|  | Non DM | | | DM | | |
|  | HR | 95% CI | *P* | HR | 95% CI | *P* |
| Model 1^†^ |  |  |  |  |  |  |
| Q2 | 0.804 | 0.587-1.102 | 0.175 | 0.757 | 0.565-1.015 | 0.063 |
| Q3 | 0.741 | 0.533-1.031 | 0.075 | 0.853 | 0.640-1.136 | 0.277 |
| Q4 | 0.535 | 0.379-0.754 | <0.001 | 0.797 | 0.599-1.062 | 0.122 |
| Model 2^‡^ |  |  |  |  |  |  |
| Q2 | 0.787 | 0.568-1.091 | 0.151 | 0.725 | 0.537-0.977 | 0.035 |
| Q3 | 0.778 | 0.550-1.100 | 0.156 | 0.824 | 0.615-1.105 | 0.197 |
| Q4 | 0.534 | 0.375-0.763 | <0.001 | 0.775 | 0.578-1.039 | 0.088 |
| Model 3^§^ |  |  |  |  |  |  |
| Q2 | 0.793 | 0.566-1.111 | 0.178 | 0.701 | 0.515-0.955 | 0.024 |
| Q3 | 0.829 | 0.579-1.188 | 0.308 | 0.859 | 0.638-1.157 | 0.319 |
| Q4 | 0.516 | 0.357-0.745 | <0.001 | 0.752 | 0.565-1.027 |  |

^†^ Model 1: crude, ^‡^Model 2: adjusted for sex, body mass index, ^§^Model 3: adjusted for sex, body mass index, serum albumin, concurrent history of hypertension, liver cirrhosis, congestive heart failure, cerebrovascular accident, History of hospitalization (within 6months)

HR = hazard ratio, LDL-C = Low density lipoprotein cholesterol, DM = diabetes mellitus

**Table S5. Hazard ratios of all-cause mortality in albumin according to LDL-C level (quartile)**

|  | Statin naive | | | | | |
| --- | --- | --- | --- | --- | --- | --- |
|  | Albumin < 4 | | | Albumin >= 4 | | |
|  | HR | 95% CI | *P* | HR | 95% CI | *P* |
| Model 1^†^ |  |  |  |  |  |  |
| Q2 | 0.863 | 0.688-1.083 | 0.206 | 0.394 | 0.200-0.778 | 0.007 |
| Q3 | 0.860 | 0680-1.087 | 0.207 | 0.617 | 0.336-1.130 | 0.118 |
| Q4 | 0.701 | 0.554-0.886 | 0.002 | 0.404 | 0.203-0.802 | 0.009 |
| Model 2^‡^ |  |  |  |  |  |  |
| Q2 | 0.816 | 0.646-1.030 | 0.088 | 0.387 | 0.193-0.776 | 0.007 |
| Q3 | 0.835 | 0.655-1.065 | 0.146 | 0.651 | 0345-1.228 | 0.185 |
| Q4 | 0.669 | 0.525-0.852 | 0.001 | 0.416 | 0.206-0.839 | 0.014 |
| Model 3^§^ |  |  |  |  |  |  |
| Q2 | 0.828 | 0.654-1.048 | 0.117 | 0.422 | 0.205-0.870 | 0.019 |
| Q3 | 0.865 | 0.677-1.107 | 0.250 | 0.715 | 0.368-1.389 | 0.323 |
| Q4 | 0.673 | 0.527-0.859 | 0.001 | 0.473 | 0.228-0.984 | 0.045 |

^†^ Model 1: crude, ^‡^Model 2: adjusted for sex, body mass index, ^§^Model 3: adjusted for sex, body mass index, concurrent history of hypertension and diabetes mellitus, liver cirrhosis, congestive heart failure, cerebrovascular accident, History of hospitalization (within 6months)

HR = hazard ratio, LDL-C = Low density lipoprotein cholesterol

**Table S6. Baseline characteristics of the study population according to history for dyslipidemia medication**

|  | No | Yes | p-value |
| --- | --- | --- | --- |
| Sex |  |  | 0.174 |
| female | 779 (56.2) | 441 (53.2) |  |
| male | 608 (43.8) | 388 (46.8) |  |
| Primary etiology |  |  | 0.000 |
| Diabetic kidney disease | 583 (42.4) | 455 (55.2) |  |
| Glomerulonephritis | 111 (8.1) | 58 (7.0) |  |
| Reno-vascular disease | 326 (23.7) | 168 (20.4) |  |
| other | 354 (25.8) | 143 (17.4) |  |
| malignancy |  |  | 0.048 |
| No | 1159 (83.6) | 721 (87.0) |  |
| Complete response | 179 (12.9) | 91 (11.0) |  |
| Keep on treatment | 33 (2.4) | 8 (1.0) |  |
| Palliative setting | 15 (1.1) | 9 (1.1) |  |
| malignancy (metastasis) |  |  | 0.369 |
| No | 1331 (96.4) | 801 (97.1) |  |
| Yes | 50 (3.6) | 24 (2.9) |  |
| Ischemic heart disease |  |  | 0.000 |
| No | 1194 (86.3) | 533 (64.3) |  |
| Yes | 190 (13.7) | 296 (35.7) |  |
| Cerbrovascular accident |  |  | 0.001 |
| No | 1151 (83.0) | 640 (77.2) |  |
| Yes | 235 (17.0) | 189 (22.8) |  |
| Congestive heart failure |  |  | 0.000 |
| No | 1187 (85.7) | 649 (78.6) |  |
| Yes | 198 (14.3) | 177 (21.4) |  |
| Atrial fibrillation |  |  | 0.973 |
| No | 1241 (89.5) | 741 (89.5) |  |
| Yes | 145 (10.5) | 87 (10.5) |  |
| Diabetes |  |  | 0.000 |
| No | 660 (47.7) | 284 (34.3) |  |
| Yes | 724 (52.3) | 544 (65.7) |  |
| Hypertension |  |  | 0.007 |
| No | 168 (12.1) | 70 (8.5) |  |
| Yes | 1217 (87.9) | 758 (91.5) |  |
| Liver cirrhosis |  |  | 0.098 |
| No | 1344 (96.9) | 813 (98.1) |  |
| Yes | 43 (3.1) | 16 (1.9) |  |
| Hospitalization history before dialysis starts |  |  | 0.305 |
| nonr | 881 (63.7) | 529 (63.8) |  |
| < 1 month | 419 (30.3) | 1. 1.6) |  |
| > 1 month | 84 (6.1) | 38 (4.6) |  |
| ACE inhibitor |  |  | 0.000 |
| No | 1342 (96.9) | 732 (93.4) |  |
| Yes | 43 (3.1) | 52 (6.6) |  |
| ARB |  |  | 0.000 |
| No | 809 (58.4) | 367 (46.2) |  |
| Yes | 577 (41.6) | 428 (53.8) |  |
| Total Cholesterol |  |  | 0.220 |
| < 200 | 1070 (88.7) | 657 (90.5) |  |
| ≥ 200 | 136 (11.3) | 69 (9.5) |  |
| Antiplatelet agents |  |  | 0.000 |
| No | 823 (59.5) | 263 (32.2) |  |
| Yes | 560 (40.5) | 554 (67.8) |  |
| Start HD access |  |  | 0.005 |
| catheter | 1140 (82.2) | 644 (77.7) |  |
| AVF | 179 (12.9) | 152 (18.3) |  |
| AVG | 63 (4.5) | 31 (3.7) |  |
| PD | 5 (0.4) | 2 (0.2) |  |
| Maintenance HD access |  |  | 0.458 |
| catheter | 324 (23.7) | 152 (18.6) |  |
| AVF | 784 (57.3) | 546 (66.8) |  |
| AVG | 257 (18.8) | 118 (14.4) |  |
| PD | 4 (0.3) | 1 (0.1) |  |
| BMI | 22.86±3.95 | 23.77±4.25 | 0.000 |
| Age at the start of dialysis | 77.76±5.42 | 77.06±5.15 | 0.002 |
| albumin (g/dL) | 3.32±0.62 | 3.38±0.61 | 0.036 |
| WBC (/mm3) | 9076.06±6414.26 | 8700.53±4676.67 | 0.114 |
| Hb (g/dl) | 9.18±1.72 | 9.32±1.52 | 0.052 |
| HDLC | 39.08±14.81 | 42.44±28.27 | 0.004 |
| LDLC | 90.80±39.74 | 84.81±45.37 | 0.013 |
| Triglycerides(mg/dl) | 117.74±70.56 | 122.67±75.70 | 0.211 |
| Calcium | 8.18±0.98 | 8.16±0.93 | 0.655 |
| Phosphate | 4.97±1.77 | 4.91±1.66 | 0.413 |
| Total Cholesterol | 146.10±46.69 | 138.65±49.22 | 0.001 |

**Table S7. Hazard ratios of all-cause mortality according to LDL-C level (quartile) using different reference cartegory**

|  | Statin naive | | |
| --- | --- | --- | --- |
|  | HR | 95% CI | *P* |
| Q3 (Reference) |  |  |  |
| Model 1^†^ |  |  |  |
| Q1 | 1.245 | 1.003-1.546 | 0.046 |
| Q2 | 0.971 | 0.780-1.209 | 0.793 |
| Q4 | 0.827 | 0.660-1.036 | 0.098 |
| Model 2^‡^ |  |  |  |
| Q1 | 1.259 | 1.007-1.574 | 0.042 |
| Q2 | 0.940 | 0.749-1.181 | 0.597 |
| Q4 | 0.812 | 0.644-1.023 | 0.077 |
| Model 3^§^ |  |  |  |
| Q1 | 1.181 | 0.939-1.484 | 0.153 |
| Q2 | 0.910 | 0.723-1.146 | 0.425 |
| **Q4** | **0.765** | **0.605-0.967** | **0.025** |

^†^ Model 1: crude, ^‡^Model 2: adjusted for gender, body mass index, ^§^Model 3: adjusted for gender, body mass index, serum albumin, concurrent history of hypertension and diabetes mellitus, liver cirrhosis, congestive heart failure, cerebrovascular accident, History of hospitalization (within 6months)

HR = hazard ratio, LDL-C = Low density lipoprotein cholesterol
